# Properties of echoic memory revealed by auditory-evoked magnetic fields

**DOI:** 10.1038/s41598-019-48796-9

**Published:** 2019-08-22

**Authors:** Tomoaki Kinukawa, Nobuyuki Takeuchi, Shunsuke Sugiyama, Makoto Nishihara, Kimitoshi Nishiwaki, Koji Inui

**Affiliations:** 1 Department of Anesthesiology, Nagoya University Graduate School of Medicine, Nagoya, 466-8550 Japan; 2 Neuropsychiatric Department, Aichi Medical University, Nagakute, 480-1195 Japan; 3 Department of Psychiatry and Psychotherapy, , Gifu University, Gifu, 501-1193 Japan; 4 Multidisciplinary Pain Center, Aichi Medical University, Nagakute, 480-1195 Japan; 5 Department of Functioning and Disability, Institute for Developmental Research, Kasugai, 480-0392 Japan; 6 Department of Integrative Physiology, National Institute for Physiological Sciences, Okazaki, 444-8585 Japan

**Keywords:** Auditory system, Attention, Neuronal physiology

## Abstract

We used auditory-evoked magnetic fields to investigate the properties of echoic memory. The sound stimulus was a repeated 1-ms click at 100 Hz for 500 ms, presented every 800 ms. The phase of the sound was shifted by inserting an interaural time delay of 0.49 ms to each side. Therefore, there were two sounds, lateralized to the left and right. According to the preceding sound, each sound was labeled as D (preceded by a different sound) or S (by the same sound). The D sounds were further grouped into 1D, 2D, and 3D, according to the number of preceding different sounds. The S sounds were similarly grouped to 1S and 2S. The results showed that the preceding event significantly affected the amplitude of the cortical response; although there was no difference between 1S and 2S, the amplitudes for D sounds were greater than those for S sounds. Most importantly, there was a significant amplitude difference between 1S and 1D. These results suggested that sensory memory was formed by a single sound, and was immediately replaced by new information. The constantly-updating nature of sensory memory is considered to enable it to act as a real-time monitor for new information.

## Introduction

According to the model by Atkinson and Shiffrin^1^, human memory is divided into three components: the sensory register, short-term store, and long-term store. The sensory register, or sensory memory, is thought to hold sensory information briefly for use in the next step of the multi-store memory system. The mechanisms of memory build-up and decay have been examined for many years^2–5^, and psychological studies have revealed the existence of brief storage^6,7^. For example, by using the method known as partial report procedures, Sperling demonstrated that subjects could remember many briefly-presented characters using short-lasting visual memory if the cue to recall the characters was given within one second after the visual display^8^. By using a similar paradigm, Darwin *et al*. demonstrated the existence of a brief storage of several seconds in the auditory system^9^. Psychological studies have revealed unique properties of sensory memory, including its decay^10^, vulnerability to interference stimuli^11,12^, and the effects of stimulus repetition^13^. However, it is not easy for psychological studies to deal with sensory memory because it is outside of our cognitive control. In addition, the duration of storage appears to be too short for standard memory and recall procedures, given that the lifetime is considered to be a few seconds^6,9,14^.

Mismatch negativity (MMN)^15–18^, a component of event-related potentials (ERPs), has been used as an objective method to observe sensory memory. When rare and frequent sensory stimuli are presented randomly, the former elicits MMN with a maximum negativity at Fz and positivity at the mastoid. MMN is considered to reflect the process of automatically detecting deviant stimuli based on short-term memory trace. Therefore, it is believed that MMN can index the sensory memory. By using MMN, for example, several researchers have measured the lifetime of sensory memory^19–22^. As the brain has to form a representation of the repetitive aspects of auditory stimulation before the occurrence of the rare stimulus for elicitation of MMN^23^, the sequence of stimulus presentation is limited.

In order to study sensory memory, we have used the change-related cortical response – a kind of event-related response that is specifically elicited when the brain detects sensory information different from the preceding sensory status^24^, and is clearly observed by magnetoencephalography (MEG) or electroencephalography. Unlike MMN, it can be elicited without repetition of a frequent stimulus^24–26^. Therefore, it is easy to use for various stimulation paradigms. Previous studies showed that the amplitude of the change-related cortical response depends on the degree of the sensory change^24,27,28^, length of the stimulus to be stored^26^, length of the preceding sensory status to be compared^28–30^, length of the decay time of the storage of previous events^26,31,32^, and the probability of the test stimulus under an oddball paradigm^33^. These findings indicate that the sensory storage and comparison processes are involved in generating the response. The storage is capable of retaining details of the sensory stimulus, like a snapshot^34^. Taken together, it appears that the storage involved in the change-related cortical response is sensory memory, according to the lifetime-based standard classification of memory^35^. The advantage of this method is that it requires no task of the subjects, and thereby subjects need not pay attention to, remember, or recall the stimulus. This allows us to observe sensory memory objectively. In this study, we used the change-related cortical response to investigate the properties of echoic memory in the brain, particularly its nature of decay. In a previous study^24^, it was shown that sensory memory acts as a real-time sensory monitor, as a single presentation of a sound was able to build up memory and affected the cortical response to the next sound. However, in order to act as a real-time monitor, there must be active forgetting, in order to update information in real-time. To clarify this, three experiments were conducted in the present study. In addition, peripheral contribution to such ERP components remain possible^36^. Therefore, we used sounds with an interaural time difference (ITD) in this study to rule out these peripheral contributions.

## Results

In the main experiment (Experiment 1), there were two sounds (Sound), lateralized to the left (Right-delay) and right (Left-delay). The sounds were labelled according to whether the sound was preceded by the same sound or different sound, and grouped into S trials and D trials. The D sounds were further grouped into 1D, 2D, and 3D, according to the number of preceding different sounds. The S sounds were similarly grouped to 1S and 2S. The effect of the preceding sound (Event) on auditory-evoked middle-latency component, N100m, was investigated in this study. The mean latency and amplitude of N100m for each condition are listed in Table 1. Three-way ANOVA showed that Sound (F_1,12_ = 12.7, p = 0.004) and Event (F_4,48_ = 25.2, p = 2.8 × 10^−11^) but not Hemisphere (F_1,12_ = 0.40, P = 0.84) were significant factors in determining the amplitude. The overall amplitude was greater for the Left-delay (L) sound (20.4 nAm) than for the Right-delay (R) sound (16.2). Although Hemisphere was not a significant factor, there was a significant Hemisphere x Sound interaction (F_1,12_ = 15.3, p = 0.002). Additionally, there was a main effect of Sound for the left hemisphere (F_1,12_ = 27.5, p = 2.1 × 10^−4^). That is, the amplitude of the response in the left hemisphere was greater for L (21.5 nAm) than for R (14.8). On the other hand, there was no significant difference between L (19.4 nAm) and R (17.7) for the right hemisphere. In other words, the contralateral bias was clear for R, but not for L. Similar findings have been reported in previous studies using MEG^37^ and fMRI^38^. These findings appear to show hemispheric differences for auditory spatial processing.

**Table 1:** Peak latency and amplitude of N100m.

	Left-delay		Right-delay	
	Left	Right	Left	Right
**Amplitude**				
1D	21.0 (7.4)	19.3 (8.8)	14.4 (3.9)	17.3 (7.2)
2D	25.1 (7.2)	21.3 (9.0)	16.5 (3.5)	18.1 (6.6)
3D	26.3 (8.1)	22.1 (10.5)	16.6 (5.0)	20.4 (8.5)
1S	17.5 (5.1)	16.3 (7.3)	12.8 (4.0)	15.8 (6.8)
2S	17.6 (6.3)	18.0 (8.5)	13.4 (3.6)	16.8 (6.8)
**Peak latency**				
1D	123.8 (14.0)	131.2 (13.7)	134.7 (18.6)	127.1 (14.6)
2D	127.3 (16.0)	130.2 (11.9)	130.2 (18.2)	121.9 (14.0)
3D	126.5 (16.1)	127.6 (15.5)	128.9 (16.0)	123.7 (17.0)
1S	123.0 (15.1)	124.8 (13.8)	128.2 (14.0)	120.0 (15.2)
2S	129.5 (20.5)	130.1 (16.7)	127.5 (15.8)	117.7 (14.7)

Data are shown as the mean (SD).

**Figure 1:**
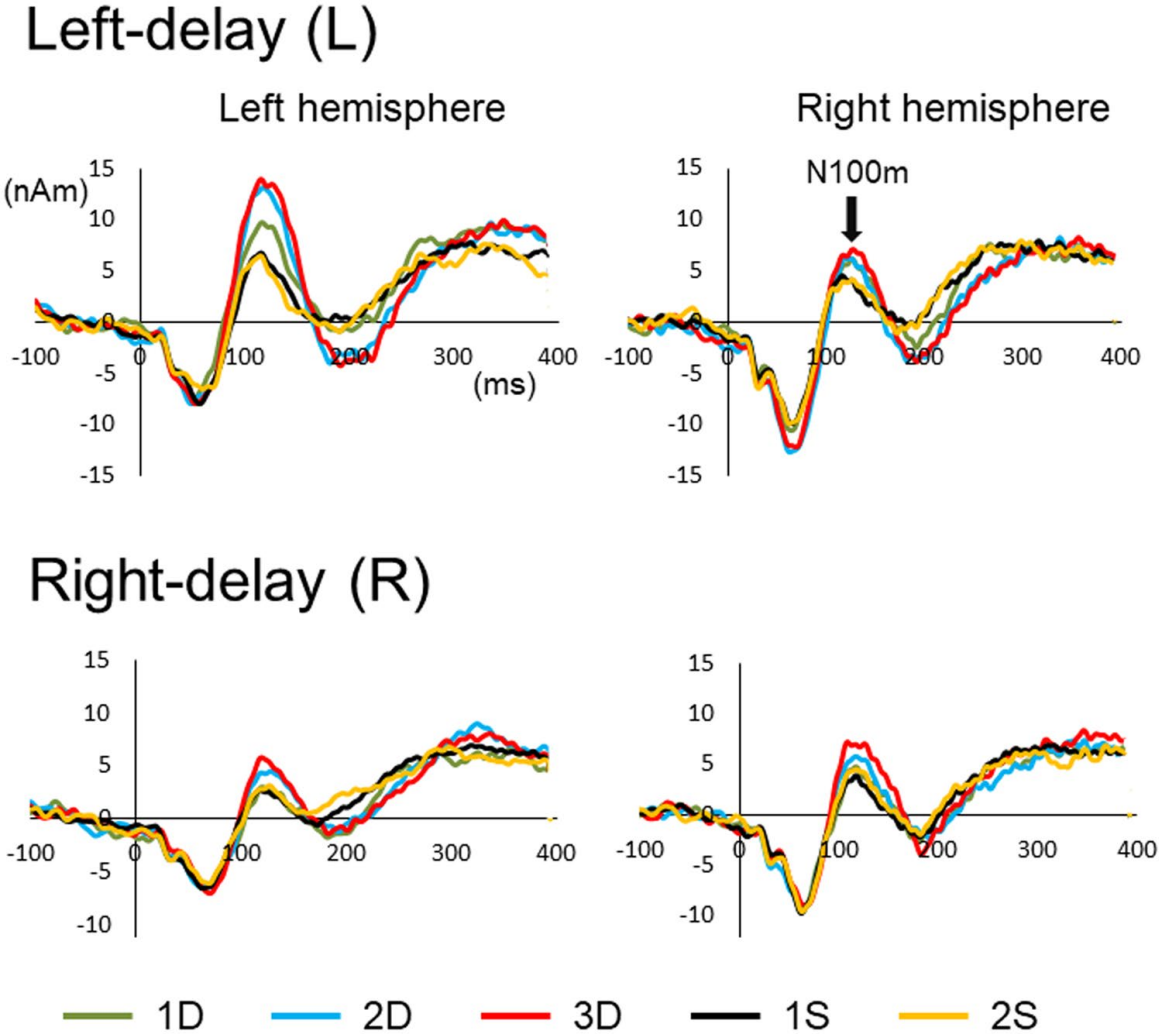
Grand-averaged waveforms of auditory-evoked cortical activity. Waveforms in response to the Left-delay sound (L) and Right-delay sound (R) are shown in the upper and lower panels, respectively. For each sound and hemisphere, there were five conditions, 1D, 2D, 3D, 1S, and 2S, which indicate how many different (D) or same (S) sounds preceded the probe sound.

As for the effects of the preceding event, the results of the post hoc tests showed that, in general, the amplitudes of D trials were greater than those of S trials: the amplitude for 1S was significantly smaller than those for 1D, 2D, and 3D; 2S was significantly smaller than 2D and 3D. It is noteworthy that the difference was significant between 1D and 1S (p = 0.039). Among the D trials, the amplitude was greater for 3D, 2D, and 1D, in that order, with significant differences between 1D and 2D (p = 0.011) and between 1D and 3D (p = 0.004). Grand-averaged waveforms of each event are shown in Fig. 1. There was no significant difference between 1S and 2S (p = 0.94). We consider that the lack of a significant difference between 1D and 2S was due to the relatively small effect of the 1D condition. A comparison between 1D and 2S with a paired t-test showed a significant difference (p = 0.004) when correction for multiple comparisons was not applied. In addition, the amplitudes for 1D and 2S are highly correlated (r2 = 0.74) with a regression line with a slope of 0.9 indicating that the amplitude is reliably greater for 1D by approximately 10%.

Regarding latency, none of the main effects were significant. Although there was a tendency for the latencies for 1S (121.0 ms) and 2S (123.1) to be shorter than those for 1D (126.1), 2D (124.3), and 3D (123.6), the difference was not significant (F_4,48_ = 2.38, p = 0.065). In contrast, there was a significant Hemisphere x Sound interaction (F_1,12_ = 16.7, p = 0.002). The results of subsequent analyses showed that in both the left (F_1,12_ = 5.01, p = 0.045) and right (F_1,12_ = 7.6, p = 0.018) hemispheres, there were significant main effects of Sound. In the left hemisphere, the latency of the response to L (122.9 ms) was shorter than that to R (126.8). In the right hemisphere, the latency for R (119.0) was shorter than that for L (125.7). That is, the peak latency of N100m was shorter and the amplitude was greater for the side with the interaural time difference (ITD) or the hemisphere contralateral to sound lateralization, confirming a previous study^39^.

**Figure 2:**
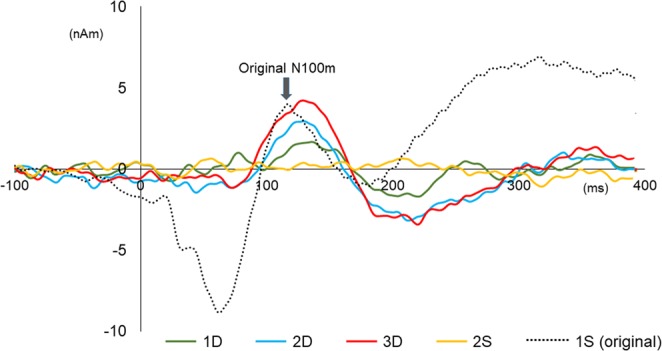
Difference waveforms with original 1S. Difference waveforms obtained by subtracting the 1S waveform are shown. Thus, the waveforms indicate cortical activity due to the presence of the prior different sounds. For comparison, the original waveform for 1S is also shown. Waveforms of both hemispheres are combined. Note the slightly later peak of the difference waveform than the original 1S waveform.

These results suggested the existence of cortical activities sensitive to the sound sequence. In order to quantify these activities, the source strength waveform of 1S was subtracted from those of other events, and the amplitude and latency were measured using the difference waveforms. Because no clear peak at around the N100m latency was seen for the subtracted 2S waveform in most of the subjects, data for the D trials were analyzed. The mean peak latencies and amplitudes are listed in Table 1. The amplitude was greater for 3D, 2D, and 1D, in that order. Three-way ANOVA showed a significant main effect of Event (F_2,24_ = 9.37, p = 0.001). Post hoc tests revealed significant differences between 1D and 2D (p = 0.002), and 1D and 3D (p = 0.012), but not between 2D and 3D (p = 0.78). The latency did not differ among events (F_2,24_ = 0.029, p = 0.97). Grand-averaged difference waveforms across hemispheres and sounds are illustrated in Fig. 2. As shown in Table 2 and Fig. 2, the N100m peak latency for the difference waveforms was slightly longer than that for the original waveforms. When the latency for the difference waveform was compared with that for the original 1S using a paired t-test, the difference was significant for 1D (p = 0.042, corrected for multiple comparisons) and 3D (p = 0.01). The latency for 2D also tended to be longer than that for the original 1S (p = 0.11).

**Table 2:** Peak latency and amplitude of difference waveforms.

	Left-delay		Right-delay	
	Left	Right	Left	Right
**Amplitude**				
1D	7.94 (3.4)	7.50 (3.9)	6.17 (2.2)	5.56 (3.6)
2D	10.2 (3.8)	9.88 (6.0)	7.79 (3.9)	6.78 (3.8)
3D	12.0 (4.2)	10.3 (7.4)	8.36 (3.81)	7.23 (3.1)
**Peak latency**				
1D	126 (25.4)	130.0 (22.7)	129.7 (23.4)	132.3 (12.1)
2D	131.3 (40.1)	134.8 (17.6)	124.5 (16.7)	124.8 (18.0)
3D	126.1 (21.0)	129.8 (17.4)	129.5 (18.6)	131.8 (20.0)

## Discussion

In the present study, we sought to clarify the properties of echoic memory by using auditory-evoked cortical responses. As the subjects did not need to pay attention to, memorize, or recall the stimuli, we could evaluate echoic memory objectively through its cortical responses. Our results revealed a significant difference in the amplitude of the evoked cortical responses for 1D and 1S, suggesting that a single presentation of the sound (R or L) was sufficient to store the information and that the storage was replaced immediately with another when the brain detected a different sound. Therefore, echoic memory can be considered to act as a monitor of the current sensory status.

### Methodological consideration: In the present study

we used the change-related cortical response^26^, which is specifically evoked when the brain detects any kind of novel sensory event. Although this response is typically evoked by an abrupt change to a continuous sensory stimulus, it is also evoked by a stimulus preceded by another with different features, after a gap^26^. In the present study, the different waveforms showed N100m peaking at 120–130 ms, which appears to correspond to the change-related N100m evoked by an abrupt change in sound location peaking at 120–135 ms^21,30,33^. The current study used a simple train of clicks as the test stimulus. As the two sounds, L and R, differed only in their phase, they were identical at each ear for every trial, which excluded the possible contribution of the periphery to the present results. The subjects’ task during the experiments was to watch a silent movie and ignore the sound, meaning that their cortical responses were automatic. Therefore, the present results suggested that the information about the sound was automatically stored in the processing pathway of the brain.

### Characteristics of echoic memory revealed in the present study

As the cortical response was significantly affected by a single event preceding the test stimulus, the information was stored during presentation of a single sound of 500 ms and was used during processing of the next sound. This indicates that the single event of the train of clicks was sufficient to retain the information for later use, which is in agreement with the instantaneous nature of sensory memory. In a psychological study by Sperling^8^, a stimulus of 25 ms was shown to be sufficient to establish visual sensory memory. In a study using dichotic listening, a single word could be stored in memory^14^. Therefore, it is reasonable that the single 500-ms sound was sufficient to be stored in an available form. However, it is significant that the present study confirmed this instantaneous nature of echoic memory without the subjects being aware of it. This relatively long duration of 500 ms was used to strengthen the storage and to reduce its decay.

The response amplitudes to 1S and 2S did not differ significantly, suggesting that once the memory for a sound was established, a change-related cortical response did not occur in response to the next sound if it was identical. As the sequences were LRR and LRRR, respectively, this means that the memory for L in these sequences was lost when the probe R was presented. This mechanism is considered to exist to avoid excessive responses to irrelevant information, and to reduce energy consumption. In contrast, repeated presentations of a sound increased the N100m amplitude following the next different sound, suggesting that memory is strengthened by repetition. Similar effects were reported in a psychological study^40^. This indicates that the present 500-ms click train was not sufficient for full storage, which is in agreement with previous findings that the amplitude of the change-related cortical response was influenced by the duration of the sensory status, when compared up to 3–6 s^30,32^. Taken together, the present results suggest that the short storage established during the presentation of a sound was replaced immediately by short storage for the next different sound. In sequences of LRL (1D) and RLL (1S), the amplitude of 1D was clearly larger than that of 1S (p = 0.039). If the storage for the first L in LRL (1D) had remained at the presentation of the next L, no change-related cortical response would have occurred. Therefore, for one auditory sub-modality, such as sound location, only one instance of the latest status is stored. In order to confirm this notion, Experiment 2 was performed in seven subjects (Supplemental Fig. 1), in which a brief click train of 50 ms composed of L or R was inserted between the original 500-ms sounds. The results showed that the brief click train had only a weak effect on processing of the next different sound; it did not elicit a significant change-related cortical response for the next sound (p = 0.15), but it could reset the memory for preceding sounds (p = 0.0026).

**Figure 3:**
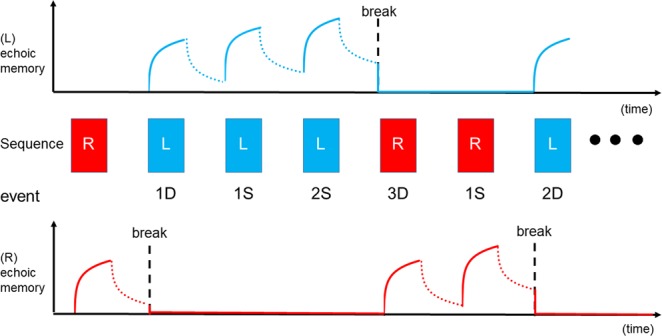
A model for echoic memory to Left-delay (L) and Right-delay (R) sounds. The Y axis indicates the strength of memory. At the breaking point, the change-related cortical response, whose amplitude is determined by the strength of the memory, is elicited.

As the occurrence probability of the two sounds was even and the storage of each sound lasted longer than the trial-trial interval in the present study, there must be a specific mechanism that shortens the memory of each sound. The results of Experiment 3 (Supplemental Fig. 2) showed that the lifetime of the memory trace in the present study was 4–6 s. Without such mechanisms, the memory would increase up to its limit during the recording, and differences among 1D, 2D, and 3D would not arise. We consider that replacement of the memory trace by new information is the main responsible mechanism. Figure 3 explains the present results using a model. In this model^24^, the strength of sensory memory is increased during stimulus presentation or by repetition of the stimulus in a positively-accelerated fashion, decreased during a blank in a negatively-accelerated fashion, and is abolished/replaced by a new stimulus.

## Conclusions

There is a debate surrounding the contributions of time-dependent decay and interference to forgetting in short-term or working memory^10^. Echoic memory, indexed by the change-related cortical response, is clearly dependent on the passage of time^26^. In the present study, a different sound preceding the probe sound was an interference stimulus, particularly the brief train in Supplemental Experiment 1. Therefore, both decay and interference contribute to forgetting in echoic memory. Under the present paradigm using only two sounds, the interference effect was powerful, almost abolishing the previous storage. It can thus be functionally regarded as replacement. The change-related cortical response is considered to be a subtype of a defense reaction^41^, playing an important role in the prompt detection of changes in the sensory environment. Sensory memory is the basis of the change-related cortical response, and therefore plays a role in survival. For this purpose, forgetting/replacement is important in order to update the current sensory status. We consider that these properties of sensory memory enable it to play a role as a real-time monitor, identifying new events to which attention may be required.

## Methods

The study protocol was designed in accordance with the Declaration of Helsinki (World Medical Association, 2008), and was approved in advance by the Ethics Committee of the National Institute for Physiological Sciences, Okazaki, Japan. All subjects provided written informed consent prior to participation. Thirteen healthy volunteers (3 women, 10 men; aged 25–55 years, mean 37 years) participated in the study. None had a history of mental or neurological disorders, nor substance abuse, in the most recent five years, and all were free of medication at the time of testing.

### Stimulation

**Figure 4:**
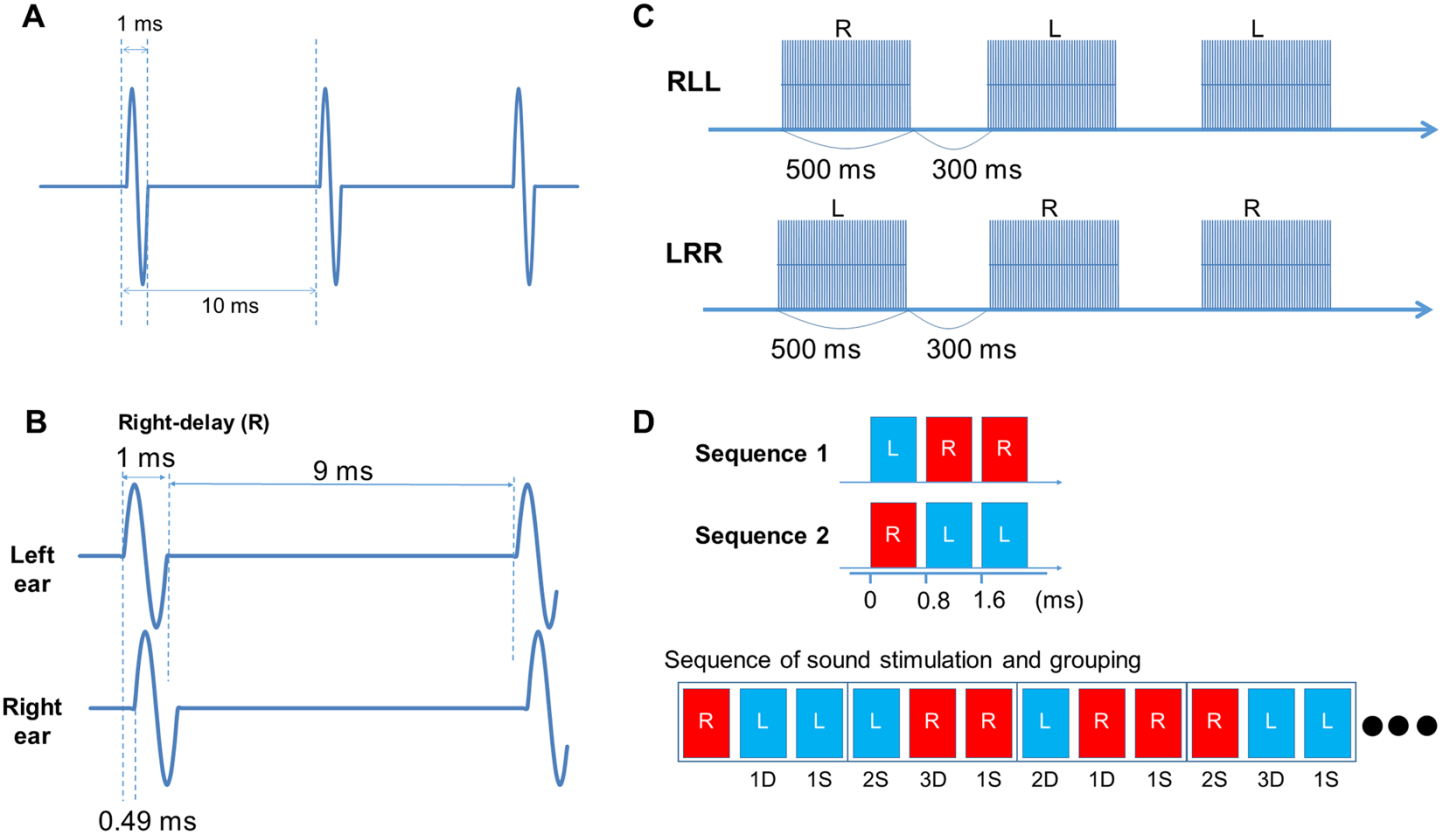
Schematic illustration of auditory stimuli. (**A**) Repetition of a 1-ms click at 100 Hz. (**B**) Left lateralized sound (R) created by inserting a 0.49-ms interaural time delay to the right side. (**C**) Two sequences consisting of RLL and LRR. (**D**) Labelling of each sound by preceding events.

The sound used in the present study was a train of clicks, 100 Hz in repetitive frequency, 70 dB SPL in sound pressure, and 500 ms in total duration. Clicks were generated as single cycles of a 1-ms sine wave (Fig. 4A,B). By inserting an ITD of 0.49 ms between sides, two sounds, Left-delay (L) and Right-delay (R), were created (Fig. 4C). Next, we made two sequences composed of LRR and RLL, as previously described^26^, with a blank of 300 ms between sounds. The two sequences, LRR and RLL, were randomly presented at an identical probability with a trial-trial interval of 800 ms during the experiment. Under this paradigm, the probability of each sound (L and R) was even. The sounds were labelled according to whether a sound was preceded by the same sound or different sound, and grouped into S trials and D trials. Among the D trials, three types appeared at an identical probability: a trial with a sound preceded by a different sound (1D), a trial preceded by two different sounds (2D) (LLR and RRL), and a trial preceded by three different sounds (3D) (LLLR and RRRL). Among the S trials, there were two types: a trial with a sound preceded by the same sound (1S), and a trial preceded by the same sound twice (2S) (LLL and RRR). Therefore, there were five types of events in this study, 1D, 2D, 3D, 1S, and 2S, with an occurrence probability of 1:1:1:2:1 for each of L and R (Fig. 4D). None of the subjects could identify the sequence of sounds even when they listened to them carefully after the experiment.

### Recordings

The subjects sat in a chair and watched a silent movie on a screen placed 2 m in front of them, and were instructed to ignore all stimuli throughout the experiment. Magnetic signals were recorded using a 306-channel whole-head type MEG system (Vector-view, ELEKTA Neuromag, Helsinki, Finland), which comprised 102 identical triple sensor elements. Each sensor element consisted of two orthogonal planar gradiometers and one magnetometer coupled to a multi-superconducting quantum interference device, and thus provided three independent measurements of the magnetic fields. In the present study, we analyzed MEG signals recorded from 204 planar-type gradiometers, which were sufficiently powerful to detect the largest signal just over local cerebral sources. Signals were recorded with a bandpass filter of 0.1–330 Hz and digitized at 1000 Hz. Analyses were conducted from 100 ms before, to 400 ms after, the onset of the stimulus. Epochs with MEG signals larger than 2.7 pT/cm were excluded from averaging. The waveform was digitally filtered with a bandpass filter of 1.0–100 Hz.

### Analysis

We performed single dipole analysis using the brain electric source analysis (BESA) software package (GmbH, Grafefling, Germany) for the main component peaking at approximately 120 ms (N100m), as previously described^24^. First, all five conditions were added for L and R. The equivalent current dipole for N100m was estimated in the auditory cortex of each hemisphere. The two-dipole model was then applied to waveforms for all conditions, and obtained source strength waveforms were used to measure the amplitude and latency of the cortical response. The latency at the point of maximum amplitude within the range of 90 to 150 ms was defined as the peak latency of N100m. The peak amplitude was defined as the difference between the peak of N100m and the polarity-reversed earlier peak at around 60 ms. This procedure minimizes problems due to a baseline shift^26^. The amplitude and latency were compared among conditions using three-way ANOVA with Sound (L and R), Hemisphere, and Event (1D, 2D, 3D, 1S, and 2S) as variables. When there was a significant difference, the amplitude and latency were compared between pairs using paired t-tests with the Bonferroni correction. In order to obtain event-specific cortical activity, the source strength waveform for 1S was subtracted from those for other events. The amplitude and latency for N100m of the subtracted waveforms were compared among the three D events by three-way ANOVA (Sound x Hemisphere x Event).

Experiments 2 and 3 were each conducted using seven subjects, in order to clarify whether a new auditory event suppresses preceding storage and to estimate the lifetime of the auditory storage under the present paradigm, respectively. The procedures of the two experiments were similar to those for the main experiment, but the sound sequence was slightly different. The methods and results are described in detail in supplementary documents (Experiment 2 and Experiment 3).

## Supplementary information

supplementary information
