# Supplementary material for: Properties of echoic memory revealed by auditory-evoked magnetic fields
Source: Sci Rep. 2019 Aug 22;9:12260. doi: 10.1038/s41598-019-48796-9 (PMC6706430; doi:10.1038/s41598-019-48796-9)
Supplement: Supplementary file 1 — supplementary information [file 41598_2019_48796_MOESM1_ESM.docx]

**Properties of echoic memory revealed by auditory-evoked magnetic fields**

Tomoaki Kinukawa^1*^, Nobuyuki Takeuchi^2^, Shunsuke Sugiyama^3^, Makoto Nishihara^4^, Kimitoshi Nishiwaki^1^, Koji Inui^5,6^

^1^Department of Anesthesiology, Nagoya University Graduate School of Medicine, Nagoya 466-8550, Japan

^2^Neuropsychiatric Department, Aichi Medical University, Nagakute 480-1195, Japan

^3^Department of Psychiatry and Psychotherapy, Gifu University, Gifu 501-1193, Japan

^4^Multidisciplinary Pain Center, Aichi Medical University, Nagakute 480-1195, Japan

^5^Department of Functioning and Disability, Institute for Developmental Research, Kasugai 480-0392, Japan

^6^Department of Integrative Physiology, National Institute for Physiological Sciences, Okazaki 444-8585, Japan

*Corresponding author

Tomoaki Kinukawa, MD

Department of Anesthesiology

Nagoya University Graduate School of Medicine,

Nagoya 466-8550,

Japan

Email: t-kinukawa@med.nagoya-u.ac.jp

**Experiment 2**

**Purpose:** To clarify whether a new auditory event suppresses preceding storage.

**Methods:** An additional experiment (Experiment 2) was conducted with seven subjects. The procedures were identical to the main experiment, but with a different sound sequence. Two sounds of 50 ms with an ITD of 0.49 ms, r and l, were used in addition to R and L. The short sounds were inserted between the original 500-ms sounds. As shown in Supplemental Fig.1A, four sequences, LlLlRr, LlLrRl, RrRrLl, and RrRrLr, were presented randomly at an identical probability. The amplitude of N100m was compared between RrRrL (or LlLlR) and RrRlL (or LlLrR), and between RlR (or LrL) and RrR (or LlL).

**Results:** Although the amplitude of N100m for LrL (33.4 ± 16.2 nAm) tended to be greater than that for LlL (31.0 ± 17.0), the difference was not significant (p = 0.15, paired t-test corrected for multiple comparisons), indicating that r and l were too short to affect the processing of the next different sound. In contrast, the amplitude of N100m was significantly greater for RrRrL than for RrRlL (p = 0.0026). Considering the above result that there was no difference between RrL and RlL, this finding suggested that the short sounds canceled the storage for preceding different sounds. If the short sound had not affected the storage, no difference should have occurred between RrRrL and RrRlL, because the above result showed that the last-but-one short sound could not affect the brain response to the last probe sound.

**Conclusion:** A sound of short duration could weaken the storage of the preceding different sound. We consider this to correspond to an interference effect of the short sound in psychological studies. This mechanism is considered to contribute to replacement of the preceding storage by a new sound.

**Supplemental Figure 1**


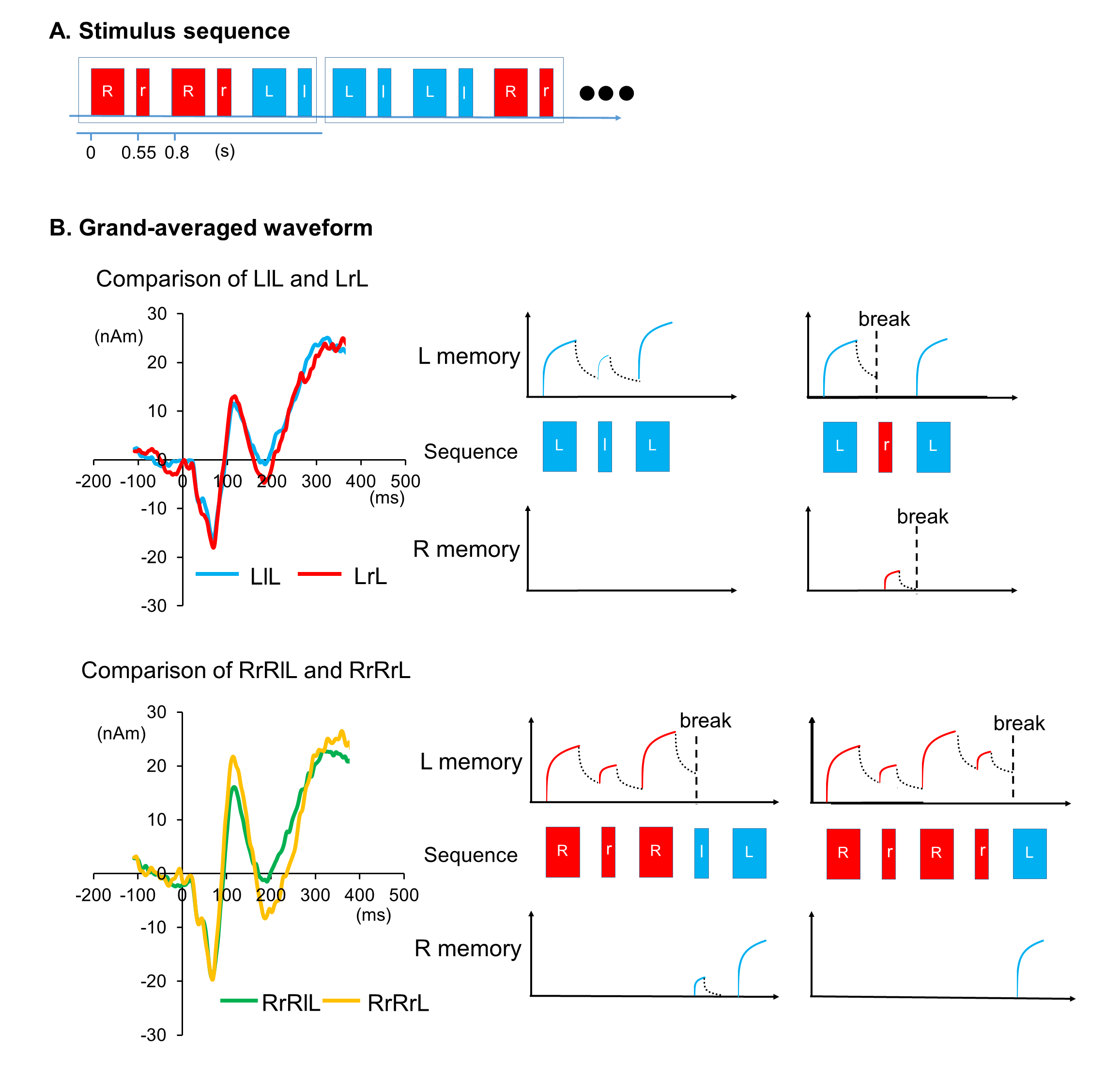


**Supplemental Fig. 1. Effects of a short sound on the change-related response.** A) schematic illustration of the stimulus sequence. B) grand-averaged waveforms of the auditory-evoked cortical activity. Comparisons of the cortical activity between LlL and LrL, and between RrRlL and RrRrL. For each sequence, the last sound is the probe sound. Right panels show an explanation of the result using a model for echoic memory to Left-delay (L and l) and Right-delay (R and r) sounds. The Y-axis indicates the strength of memory. These results suggested that, at the breaking point, the memory for preceding different sounds is reduced.

**Experiment 3**

**Purpose:** To estimate the lifetime of echoic memory in the present study.

**Methods:** Seven subjects participated. Procedures were identical to the main experiment, but with a different sound sequence, as shown in Supplemental Fig. 2A. There were five stimulus conditions with different inter-stimulus intervals (ISIs); 0.5, 1, 2, 4, or 6 s. Recordings for each condition were made in different blocks, with the order randomized among subjects. For each condition, brain responses to 1D and 1S sounds were analyzed, and the N100m amplitude was compared between 1D and 1S. When the memory of a preceding sound remained, the next 1D sound was expected to elicit change-related responses, i.e., larger responses for 1D than 1S. The difference was analyzed using two-way ANOVA for each condition with Hemisphere and Sound as variables. As the change-related response had a slightly longer peak latency than the onset N100m, a comparison using peak latency might not be appropriate. Therefore, the significance of the amplitude difference was also analyzed using difference waveforms. The difference waveform was obtained by subtracting the waveform for 1S from that for 1D. The N100m amplitude of the difference waveform was then measured and expressed as multiples of the standard deviation (SD) of the pre-stimulus baseline. We defined N100m to be significant in each condition for each subject when the response was larger than 4 SD of the baseline.

**Results:** Grand-averaged original and difference waveforms for each condition are shown in Supplemental Fig. 2B. The amplitude of N100m for 1D was significantly greater than that for 1S for the 0.5 s (F_1,6_ = 23.2, p = 0.03), 1 s (F_1,6_ = 10.2, p = 0.02), and 2 s (F_1,6_= 9.9, p = 0.02) conditions, but not for 4 s (p = 0.39) and 6 s (p = 0.12). Analyses of the difference waveforms revealed significant responses (larger than 4 SD of the baseline) in 11, 9, 7, 10, and 4 hemispheres out of 14, for the 0.5, 1, 2, 4, and 6 s conditions, respectively.

**Discussion:**

Although the N100m amplitude of the original waveform did not differ significantly between 1D and 1S for the 4 s condition, there was a significant response in the difference waveform in 10 out of 14 hemispheres in this ISI condition. The reason for the discrepancy appeared to be the finding that the change-related response peaked slightly later than the onset response (Supplemental Fig. 2B). In contrast, the analyses using the difference waveform showed a significant response in only four hemispheres for the 6 s condition, in addition to the lack of significant difference in the original waveform. Based on these results, we considered that the lifetime of echoic memory in the present study was approximately 4–6 s, which was longer than the interval from the onset of a sound to the onset after the next sound in the main experiment.

**Supplemental Fig. 2**

**
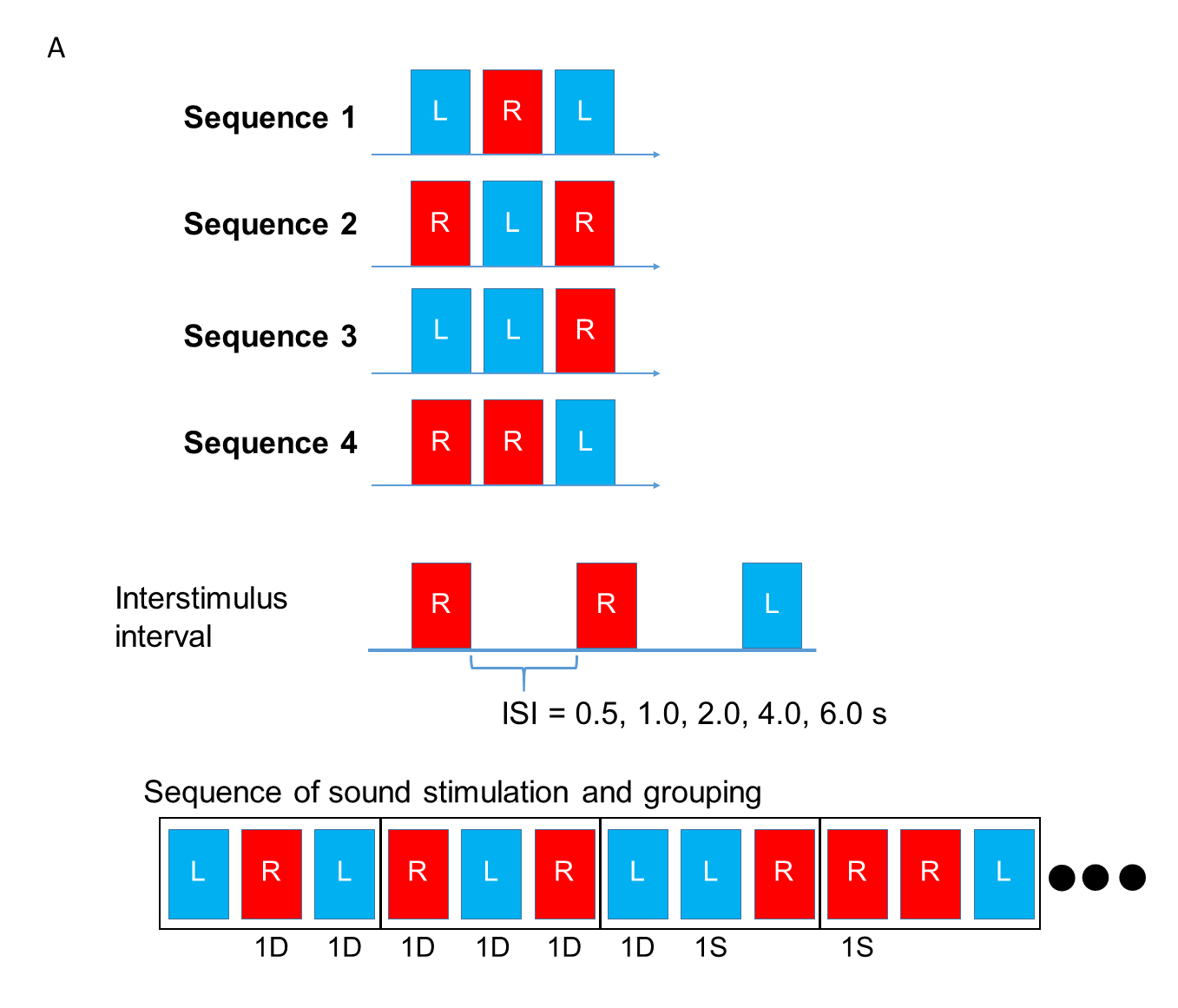
**

**Supplemental Fig. 2A. Lifetime of echoic memory.** A) Schematic illustration of the stimulus sequence. We made four fixed sequences, LRL, RLR, LLR, and RRL, and these were presented randomly with equal probability. Under this stimulus paradigm, approximately half of the sounds were 1D or 1S. The interstimulus interval (ISI) was manipulated from 0.5 to 6 s.

**
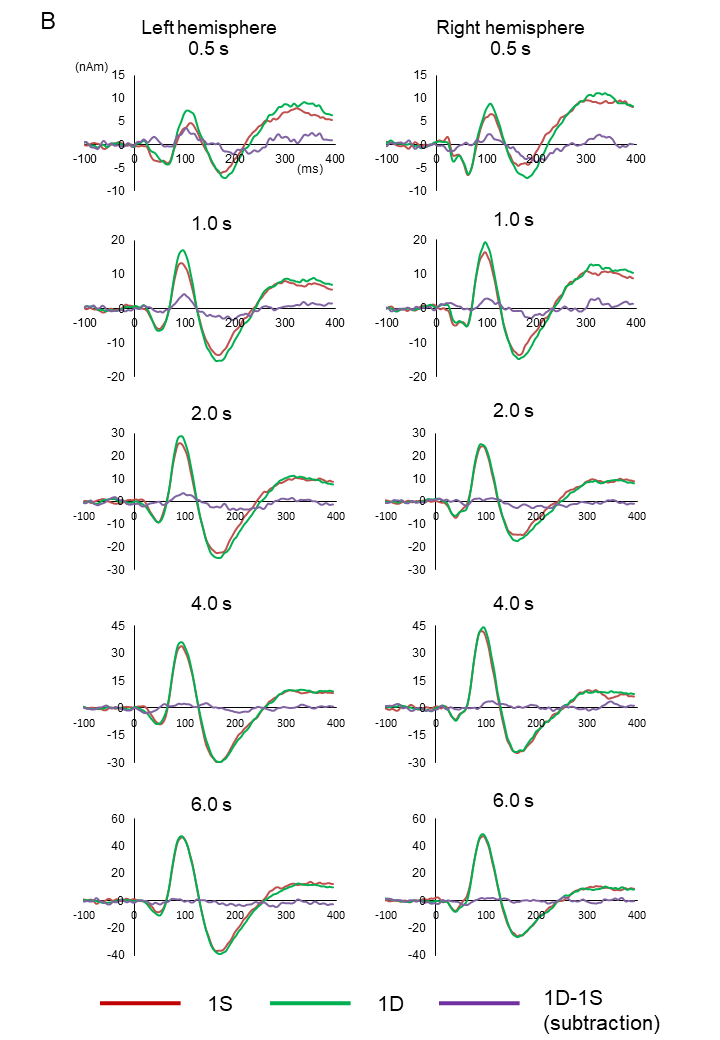
**

**Supplemental Fig. 2B. Grand-averaged original and difference waveforms.** Original and difference waveforms for each ISI condition are shown. Left and right panels show the cortical activity of left and right hemispheres, respectively. Note the greater amplitude of the 1D response in the 0.5, 1, and 2 s conditions.
